# Medication compliance of children with epilepsy: a cross-sectional survey

**DOI:** 10.1186/s13052-023-01525-5

**Published:** 2023-11-16

**Authors:** Shanshan Wang, Xu Zhang, Yongqian Wang, Jinfang Zhou

**Affiliations:** Department of Neurology, Children’s Hospital of Nanjing Medical University, No. 72, Guangzhou Road, Gulou District, Nanjing, Jiangsu Province China

**Keywords:** Medication compliance, Children, Epilepsy, Treatment, Nursing, Care

## Abstract

**Background:**

Good medication compliance is very important for the prognosis of children with epilepsy. We aimed to evaluate the status and influencing factors of medication compliance in children with epilepsy and to provide insights to the clinical nursing care of children with epilepsy.

**Methods:**

We selected epileptic children admitted to Children’s Hospital of Nanjing Medical University from February 1, 2022 to August 31, 2022. Self-designed questionnaire and medication compliance scale were used to evaluate the characteristics and medication compliance of children with epilepsy. Pearson correlation analysis and multivariate logistic regression were used to analyze the influencing factors of medication compliance.

**Results:**

A total of 156 children with epilepsy were included, the incidence of poor compliance in children with epilepsy was 37.18%. Pearson correlation analysis indicated that age(r = 0.622), courses of epilepsy(r = 0.553), parental education level(r = 0.506), monthly household income(r = 0.652) and number of drugs taken(r = 0.577) were correlated with the compliance(all P<0.05). Logistic regression analyses indicated that age ≤ 6 y(OR = 2.104, 95%CI: 1.712 ~ 2.527), courses of epilepsy ≤ 3 years(OR = 2.661, 95%CI: 2.089 ~ 2.941), low parental education level(OR = 1.977, 95%CI: 1.314 ~ 2.351), monthly household income ≤ 5000 RMB(OR = 2.812, 95%CI: 2.194 ~ 3.181), number of drugs taken ≥ 3(OR = 3.025, 95%CI: 2.336 ~ 3.475) were the influencing factors of medication compliance in children with epilepsy(all P<0.05).

**Conclusions:**

The medication compliance of children with epilepsy needs to be improved, and the medication compliance of children is affected by age, courses of epilepsy, parental education level, monthly household income and number of drugs taken. Clinical medical personnel take targeted nursing measures against these factors to improve the medication compliance of children with epilepsy.

**Supplementary Information:**

The online version contains supplementary material available at 10.1186/s13052-023-01525-5.

## Background

Epilepsy is a brain disease characterized by a persistent tendency to seizures and the neurobiological, cognitive, psychological and social consequences of the disease. Epilepsy is considered to be two or more unexplained seizures in more than 24 h. Children with epilepsy, especially infants, are different not only in clinical manifestations of epilepsy from adults, but also in etiology and response to antiepileptic drugs [1, 2]. The recurrent abnormal discharge of neurons in the brain leads to sudden and temporary brain dysfunction [2, 3]. Its clinical manifestations are dysfunction of consciousness, movement, sense, spirit or autonomic nerves [4]. It is reported that nearly 50 million people worldwide have been diagnosed with epilepsy, and the number is increasing at the rate of about 2 million every year, of which 50% are children, and 10.5 million children under 15 years old have epilepsy [5, 6]. Epilepsy has an increased risk of mortality (twice or more than standardized mortality in the general population) and sudden death [7]. In China, 60% of epilepsy patients originated in childhood, and the incidence rate of children is 151/100,000 [8, 9]. Epilepsy is easy to recur and the course of the disease is relatively long, which can seriously affect the quality of life of children in the growth and development period [10]. Therefore, it is of great clinical significance to effectively control the occurrence and development of childhood epilepsy.

At present, the main treatment of epilepsy is to take antiepileptic drugs reasonably, regularly and for a long time. As a common treatment method for epilepsy control, antiepileptic drugs can effectively control seizures in 70% of newly diagnosed epilepsy patients, but poor drug compliance is an important challenge for effective treatment of epilepsy patients and a major factor for treatment failure [11–13]. An important part of the management of people with epilepsy is to educate families and children about the disease, its management, its harm and how to deal with it. It is important to build a successful treatment alliance. In some cases, educational and psychological assessments may be necessary to assess possible learning disabilities or abnormal behavior patterns that may coexist with epilepsy [14]. It has been reported that 1/4 ~ 2/3 of epileptic patients have shown drug non-compliance [15]. The long-term treatment of epilepsy is mainly based on oral antiepileptic drugs, but some children have poor compliance, and are prone to self withdrawal, change of dosage, failure to take drugs in time, wrong dosage and other behaviors, leading to poor prognosis and prolonged course of disease [16–18]. Therefore, this study aimed to analyze the current situation and influencing factors of children’s compliance with antiepileptic drugs, provide evidence support for the nursing and treatment of children with clinical epilepsy, so as to improve children’s compliance with medication and improve the prognosis of children with epilepsy.

## Methods

### Ethics

This study was a cross sectional survey. The study protocol had been approved by the ethical committee of our hospital (approval number: 220,145-a). And written informed consents had been obtained from all the guardians and children.

### Study population

This study selected epileptic inpatient children admitted to our hospital(a tertiary children hospital in Nanjing, China) from February 1, 2022 to August 31, 2022 as the research populations. The inclusion criteria of children were: (1) The age of children was less than 16 years old; (2) The child was diagnosed as epilepsy according to the diagnostic criteria of the International Anti-epileptic Alliance;(3) Children and guardians were fully informed and voluntarily agreed to participate in the study. The exclusion criteria for children were: (1) children with medication time less than 1 month; (2) In addition to antiepileptic drugs, children who took other drugs that affect the nervous system; (3) Children who themselves or their guardians were unwilling to participate in this study.

Survey tools.

This study used self-designed questionnaire and compliance scale. First, we used the general information questionnaire to collect the general data, including age, gender, body mass index (BMI), whether the child was the only child of family, place of residence, guardians, courses of epilepsy, parental education level, monthly household income (RMB), number of drugs taken and the number of convulsions in recent past year. We have assessed both mothers’ and fathers’ education level in this study, and we included the highest education level for analysis. In addition, we investigated the medication compliance of children with Morisky Medication Adherence Scale-8 (MMAS-8) [19, 20]: the scale was compiled by Morisky D E et al. in 1986, including 4 questions, and 4 questions were added in 2008. The answer to “Never” was scored 1 point, “Occasionally” was scored 0.75 points, “Frequently” was scored 0.5 points, “very common” was scored 0.25 points, and “All the time” was scored 0 points. The total score of MMAS-8 scale was 0 ~ 8, the score < 6 indicated poor medication compliance, and the score ≥ 6 indicated good medication compliance of children.

### Data collection

All survey form collectors have received unified training in advance. We strictly abided by the principles of voluntariness, equality and confidentiality, explained the importance and significance of the study to the children and their guardians in detail before collecting the questionnaire, and collect data by asking the children to fill in the questionnaire or the parents to fill in the questionnaire after obtaining the consent and cooperation of the children and their families. In the process of the survey, we used unified professional guiding language to give appropriate explanations to the items and questions that parents did not understand one by one. Each questionnaire was guaranteed to be completed for no less than 10 min. After the questionnaire was completed, it was recovered on the spot and the integrity of the questionnaire was checked. If any omission or missing was found, the family members were encouraged to fill in the missing items on the basis of not violating the principle of voluntariness.

### Statistical analysis

In this study, SPSS 23.00 software was used for data analysis. The measurement data were described by mean ± standard deviation. The t-test/Kruskal Wallis nonparametric rank sum test was used for inter group comparison. Classified variable data were described by constituent ratio, and chi square test was used for comparison between groups. Pearson analysis and multivariate logistic regression were used to analyze the influencing factors of medication compliance. In this survey, P<0.05 was taken as the difference between groups with statistical significance.

## Results

### The characteristics of children

A total of 156 children with epilepsy were included, of whom 58 children scored MMAS-8 < 6 with poor compliance, the incidence of poor compliance in children with epilepsy was 37.18%. The characteristics of 156 children are presented in Table 1. The youngest child included was two years old, and the oldest was 15 years old. As indicated in Table 2, there were significant differences in the age, courses of epilepsy, parental education level, monthly household income and number of drugs taken between children with poor and good compliance(all P<0.05). No significant differences in the gender, BMI, only child of family, place of residence, guardians and number of convulsions in the past year between children with poor and good compliance (all P > 0.05).

**Table 1:** The characteristics of children(n = 156)

Variables	Cases/mean ± standard deviation
Age(y)	7.68 ± 2.55
Male/female	87/69
BMI (kg/m^2^)	20.694 ± 2.12
Place of residence	
Rural area	71
City	85
Guardians	
Parents	107
Grandparents	44
Others	5
Courses of epilepsy(years)	2.97 ± 1.44
Monthly household income (RMB)	4108.22 ± 515.61
Number of drugs taken	
1	44
2	56
≥ 3	56
Number of convulsions in the past year	
1 ~ 2	91
3 ~ 5	52
≥ 6	13

**Table 2:** The characteristics comparision of children with different compliance(n = 156)

Variables	Poor compliance children(n = 58)	Good compliance children(n = 98)	t/F	P
Age(y)	6.16 ± 2.33	10.02 ± 2.85	2.119	0.023
Male/female	32/26	55/43	1.215	0.086
BMI (kg/m^2^)	20.24 ± 2.51	21.01 ± 3.14	3.544	0.104
Only child of family			1.507	0.079
Yes	36(62.07%)	59(60.20%)		
No	22(37.93%)	39(39.80%)		
Place of residence			1.339	0.142
Rural area	26(44.83%)	45(45.92%)		
City	32(55.17%)	53(54.08%)		
Guardians			1.284	0.166
Parents	40(68.97%)	67(68.37%)		
Grandparents	16(27.59%)	28(28.57%)		
Others	2(3.44%)	3(3.06%)		
Courses of epilepsy(years)	2.67 ± 1.13	3.88 ± 1.42	1.292	0.023
Parental education level			1.946	0.041
High school	41(70.69%)	16(16.33%)		
Junior college	12(20.69%)	66(67.35%)		
Undergraduate	3(5.17%)	12(12.24%)		
Master or doctor	2(3.45%)	4(4.08%)		
Monthly household income (RMB)			2.755	0.016
≤ 5000	49(84.48%)	42(42.86%)		
> 5000	9(15.52%)	56(57.14%)		
Number of drugs taken			1.092	0.011
1	4(6.90%)	40(40.82%)		
2	15(25.86%)	41(41.83%)		
≥ 3	39(67.24%)	17(17.35%)		
Number of convulsions in the past year			1.285	0.107
1 ~ 2	33(56.90%)	58(59.19%)		
3 ~ 5	20(34.48%)	32(32.65%)		
≥ 6	5(8.62%)	8(8.16%)		

BMI, body mass index

### Pearson correlation analysis

As showed in Table 3, Pearson correlation analysis indicated that age(r = 0.622), courses of epilepsy(r = 0.553), parental education level(r = 0.506), monthly household income(r = 0.652) and number of drugs taken(r = 0.577) were correlated with the compliance(all P<0.05). The younger the children, the longer the duration of epilepsy, the lower the education level of their parents, the lower the monthly income of the family, the more types of drugs they take, and the worse their compliance may be.

**Table 3:** Pearson correlation analysis of medication compliance and characteristics of children

Variables	r	P
Age(y)	0.622	0.041
Gender	0.127	0.104
BMI (kg/m^2^)	0.113	0.092
Only child of family	0.121	0.088
Place of residence	0.201	0.115
Guardians	0.148	0.074
Courses of epilepsy(years)	0.553	0.038
Parental education level	0.506	0.016
Monthly household income (RMB)	0.652	0.021
Number of drugs taken	0.577	0.026
Number of convulsions in the past year	0.116	0.104

BMI, body mass index

### Influencing factors of medication compliance in children with epilepsy

The variable assignments of multivariate logistic regression were presented in Table 4. As indicated in Table 5, logistic regression analyses indicated that age ≤ 6 y(OR = 2.104, 95%CI: 1.712 ~ 2.527), courses of epilepsy ≤ 3 years(OR = 2.661, 95%CI: 2.089 ~ 2.941), low parental education level(OR = 1.977, 95%CI: 1.314 ~ 2.351), monthly household income ≤ 5000 RMB(OR = 2.812, 95%CI: 2.194 ~ 3.181), number of drugs taken ≥ 3(OR = 3.025, 95%CI: 2.336 ~ 3.475) were the influencing factors of medication compliance in children with epilepsy(all P<0.05).

**Table 4:** The variable assignments of multivariate logistic regression

Variables		Assignment
Non-compliance	Y	Yes = 1, no = 2
Age(y)	X_1_	≤ 6 = 1, >6 = 2
Courses of epilepsy(years)	X_2_	≤ 3 = 1, >3 = 2
Parental education level	X_3_	High school = 1, junior college = 2, undergraduate = 3, master or doctor = 4
Monthly household income (RMB)	X_4_	≤ 5000 = 1, >5000 = 2
Number of drugs taken	X_4_	≥ 3 = 1, 2 = 2, 1 = 3

**Table 5:** Logistic regression analysis on the influencing factors of medication compliance in children with epilepsy

Variables	β	Wald	OR	95%CI	P
Age ≤ 6 y	0.101	0.185	2.104	1.712 ~ 2.527	0.013
Courses of epilepsy ≤ 3 years	0.114	0.169	2.661	2.089 ~ 2.941	0.008
High school of parental education level	0.117	0.199	1.977	1.314 ~ 2.351	0.021
Monthly household income ≤ 5000 RMB	0.123	0.171	2.812	2.194 ~ 3.181	0.011
Number of drugs taken ≥ 3	0.109	0.178	3.025	2.336 ~ 3.475	0.009

## Discussions

At present, the prevalence of epilepsy is not optimistic. The development of prevention and control of epilepsy still mainly depends on drug treatment, and long-term oral drugs are mainly used [21]. This requires that children have good medication compliance, so as to ensure the safety and effectiveness of medication [22, 23]. To improve the compliance of children with drug use is one of the key tasks of clinical health care providers. Finding the key points to improve the compliance of children with drug use can provide effective measures. Based on the results of this survey, we have found that the incidence of poor compliance in children with epilepsy is 37.18%, for children with age ≤ 6 y, courses of epilepsy ≤ 3 years, low parental education level, monthly household income ≤ 5000 RMB, number of drugs taken ≥ 3, they may have higher risk of poor medication compliance in children with epilepsy, early prevention and nursing intervention measures should be taken for these children to improve the medication compliance of children.

In this study, 37.18% of the children have poor medication compliance, and their medication compliance needs to be improved, which is consistent with the results of many previous studies [24, 25]. The compliance of older children is significantly higher than that of younger children. 100 epileptic children aged 2 to 14 years were investigated in a cross-sectional survey for medication compliance, which have shown that only 28.00% of the children can fully comply with the prescription [26], the difference may be associated with the different areas and populations between studies, Besides, we inlcuded some 14 ~ 15 years aged children, which may have higher compliance. According to the study on medication compliance of 106 outpatients or inpatients with epilepsy, 66.98% of the children have medication non-compliance behaviors, such as drug reduction or withdrawal without the consent of the doctor when the condition has improved consciously, or drug change and drug refusal when the condition changed [27]. Because epilepsy is easy to recur, regular follow-up is required. During the long-term treatment, the understanding of the disease gradually increases, and most guardians and children can take medicine according to the doctor’s advice [28, 29]. However, when the patient’s condition is stable, there may be self medication reduction and drug withdrawal due to insufficient understanding of the disease and drugs and weak awareness of drug use according to the doctor’s advice [30]. It is suggested that in the follow-up medication education for patients, emphasis should be placed on the importance of timely, dose based and frequency based medication, especially the management of timely medication and self adjustment of medication [31, 32].

We have not found the difference on the BMI, gender, place of residence, the only child of the family, courses of epilepsy, and guardians between children with good and poor medication compliance. Previous study [33] has also found that BMI and gender are not the influencing factors of medication compliance, which is consistent with our findings. Some studies [34, 35] have pointed out that the medication compliance of children living in cities is better than that of children living in rural areas, which may be related to the higher living standards of urban children and the relatively higher income and education level of their parents. We did not find this difference, which may be related to the small sample size included in the study. The duration of epilepsy is significantly related to medication compliance. The longer the course of epilepsy is, the better the compliance is. This finding is consistent with the results of previous studies [36, 37], and the reason may be that with the prolongation of the course of the disease, the higher the attention and cognitive level of the disease, the higher the compliance with medication.

The role of guardians in medication compliance of children is particularly important. Therefore, in the process of chronic disease management for children, it is necessary to clarify the importance of guardians, promote the establishment of a positive relationship with children, and improve the participation of guardians and encourage children to improve drug compliance [38, 39]. The reason why most children’s medication compliance is low is that they forget to how to take the medication. The guardian can set alarm clock prompts, memos, etc. to remind children in time to use drugs. During the illness period, the children may have psychological changes, such as social disorder and depression, due to suspension from school or inability to engage in exercise [40]. The guardian should provide more psychological support, such as praise and reward for the children after medication, to help them establish a positive attitude, and their medication compliance will also be improved [41, 42].

Family income and parents’ education level have significant influence on children’s compliance. Previous studies [43–45] reported that family economic factors affected the compliance of children with drug use. Families with good economic conditions are able to bear the medical expenses of patients and can insist on treatment, and the effect is significant to improve compliance [46]. The educational level of parents affects the compliance of children with medication. The proportion of non-compliance behavior of parents with low educational background is high, which is significantly different from that of families with relatively high educational background. The compliance of children with parents with low educational level is relatively poor. Parents lack knowledge about epilepsy, and epilepsy has a long course and complex manifestations. If children and parents lack relevant knowledge or listen to ancestral secret prescriptions, they will often give up systematic and regular drug treatment [47, 48]. Therefore, parents of children should improve their cognitive ability and knowledge about epilepsy. Through health education, children and parents can be explained about the mechanism of epilepsy, its harm, and the clinical value and necessity of antiepileptic treatment, make them realize that epilepsy is a disease that can be treated, rational medication can reduce or prevent recurrent seizures, children can learn and grow normally, so that they can correctly take epilepsy treatment and nursing care [49].

The more complex the antiepileptic drugs taken, the worse the compliance of the children. The more kinds of drugs, the more reluctant children may be to take drugs. Besides, some drugs require frequent blood drug level monitoring to adjust drug use strategies. [50]. Drug treatment should be based on the type of seizures and other individual factors. Epileptic syndrome is a disease characterized by one or more specific types of epilepsy, with a specific age of onset and prognosis. Because each patient has many choices, choosing which drug to use is always a personalized decision based on comprehensive considerations [51]. Therefore, reasonable drug treatment and category selection are of great significance to the medication compliance of children.

How to improve the medication compliance of children with epilepsy is of great significance to the prognosis of seizure children. If clinicians lack adequate communication with children and parents, and do not formulate corresponding individualized treatment plans, they are unclear about the disease and drug guidance, children and parents do not understand the doctor’s orders, and their compliance decreases. The type of seizures had a significant influence on adhering to medication, but in China, most clincial diagnosis will not give the clear and detailed types of seizures.Therefore, it is necessary to strengthen the communication between doctors and their families, fully communicate with children and parents, and provide explanations, encouragement and support [52–54]. If necessary, parents should be invited to participate in the development of treatment plans. Regular telephone follow-up can continuously remind children to pay attention to the importance of taking medicine according to the doctor’s advice. When conditions permit, special outpatient clinics for children with epilepsy should be opened to actively treat children with epilepsy. Children and parents who lack adequate understanding of adverse reactions often change their dosage or even stop taking medicine. We should implement medication guidance, fully understand the adverse reactions of drugs when formulating treatment plans, and try to use single drug treatment. The dosage of antiepileptic drugs varies greatly among individuals, so it is better to make decisions according to the effective blood concentration [55]. Besides, heath care providers should pay attention to family support and the role of family members in the treatment, strive for the cooperation of parents, and form a treatment and nursing model combining hospital, children and family [56].

There are some deficiencies in this study that deserve consideration. First of all, the children with epilepsy collected in this study are all from a tertiary hospital, and the sample size of children collected is small. Secondly, there may be other factors that affect the compliance of children with drug use that are not included in the analysis. Some probable influencing factors on medication compliance such as comorbid conditions (like cerebral palsy and mental retardation), types of seizure, types of treatment drugs should be included for further analysis. Future researches choose more hospitals of different levels, extend the collection time, increase the number of samples collected are needed, so as to obtain more accurate research results, and provide evidence support for the treatment and nursing care of children with epilepsy.

## Conclusions

To clarify the current status of drug compliance and related factors in children with epilepsy has important reference value for the formulation of reasonable and effective coping strategies. In this study, we have found that the incidence of poor compliance in children with epilepsy is 37.18%. Age ≤ 6 y, courses of epilepsy ≤ 3 years, low parental education level, monthly household income ≤ 5000 RMB, number of drugs taken ≥ 3 are the influencing factors of medication compliance in children with epilepsy. Targeted nursing measures should be taken to improve the medication compliance of children with epilepsy. Future studies should perform more investigations on the role of some probable influencing factors such as comorbid conditions, types of seizure, types of treatment drugs on the medication compliance of epilepsy children, and the reports on the medication compliance of epilepsy children from different population and areas are needed.

### Electronic supplementary material

Below is the link to the electronic supplementary material.

Supplementary Material 1

## Data Availability

All data generated or analyzed during this study are included in this published article.

## Abbreviations

BMI: Body mass index
MMAS-8: Morisky Medication Adherence Scale-8
