# Supplementary material for: Medication compliance of children with epilepsy: a cross-sectional survey
Source: Ital J Pediatr. 2023 Nov 16;49:153. doi: 10.1186/s13052-023-01525-5 (PMC10655363; doi:10.1186/s13052-023-01525-5)
Supplement: Supplementary file 1 — Supplementary Material 1 [file 13052_2023_1525_MOESM1_ESM.docx]

Dear editor:

Manuscript ITJP-D-23-00078 entitled “Medication compliance of children with epilepsy: A cross-sectional survey", which we submitted to Italian Journal of Pediatric, has now been revised and resubmitted, the responses to the comments of the reviewers are organized one by one at the bottom of this letter.

We sincerely thank you for your tireless patience and reviewer’s wise comments on our manuscript, we marked our responses in red color to distinct from the reviewers’ suggestion, and marked the revised parts in the manuscript with red color, sincerely hope this will simplify your work.

If you have any questions, please do not hesitate to contact us.

Best regards,

Zhou

Reviewer reports:

none sence...no subject verb agrement

Thank you, we have corrected the title as you kindly suggested, please see the revised title.

what do you mean rational medication, is there any irrational medication..?

Thank you for your discreet consideration, we have revised this sentence to make it more clear, please see the revised abstract.

add prefix and

Thank you, we have added “and” as you kindly suggested, please see the revised abstract section.

Please specify the name of the hospital

Thank you, we have specified the name of the hospital as you kindly suggested, please see the revised abstract.

please delete it

Thank you, we have deleted it as you kindly suggested, please see the revised abstract.

please add the 95% CI

Thank you for your kind suggestion, we have added the 95% CI as you kindly suggested, please see the revised abstract.

you need to mention those strong factors at least

Thank you, we have explained the factors as you kindly suggested, please see the revised abstract.
